# Operative Difficulty, Morbidity and Mortality Are Unrelated to Obesity in Elective or Emergency Laparoscopic Cholecystectomy and Bile Duct Exploration

**DOI:** 10.1007/s11605-022-05344-7

**Published:** 2022-05-31

**Authors:** Ahmad H. M. Nassar, Khurram S. Khan, Hwei J. Ng, Mahmoud Sallam

**Affiliations:** 1 Laparoscopic Upper GI and Biliary Service, University Hospital Monklands, Airdrie, Scotland, UK; 2 Department of Surgery, Glasgow Royal Infirmary, Glasgow, Scotland, UK; 3 Department of Surgery, Royal Alexandra Hospital, Paisley, Glasgow, Scotland, UK

**Keywords:** Morbid obesity, Body mass index (BMI), Benign biliary surgery, Laparoscopic cholecystectomy, Common bile duct exploration, Difficulty grading

## Abstract

**Objectives:**

The challenges posed by laparoscopic cholecystectomy (LC) in obese patients and the methods of overcoming them have been addressed by many studies. However, no objective tool of reporting operative difficulty was used to adjust the outcomes and compare studies. The aim of this study was to establish whether obesity adds to the difficulty of LC and laparoscopic common bile duct exploration (LCBDE) and affects their outcomes on a specialist biliary unit with a high emergency workload.

**Methods:**

A prospectively maintained database of 4699 LCs and LCBDEs performed over 19 years was analysed. Data of patients with body mass index (BMI) ≥ 35, defined as grossly obese, was extracted and compared to a control group.

**Results:**

A total of 683 patients (14.5%) had a mean BMI of 39.9 (35–63), of which 63.4% met the definition of morbidly obese. They had significantly more females and significantly higher ASA II classifications. They had equal proportions of emergency admissions, similar incidence of operative difficulty grades 4 or 5 and no open conversions and were less likely to undergo LCBDE than non-obese patients. There were no significant differences in median operative times, morbidity, readmission or mortality rates.

**Conclusions:**

This study, the first to classify gall stone surgery in obese patients according to operative difficulty grading, showed no difference in complexity when compared to the non-obese. Refining access and closure techniques is key to avoiding difficulties. Index admission surgery for biliary emergencies prevents multiple admissions with potential complications and should not be denied due to obesity.

**Supplementary Information:**

The online version contains supplementary material available at 10.1007/s11605-022-05344-7.

## Introduction

World Health Organisation statistics estimate that obesity has nearly tripled worldwide since 1975^1^. The percentage of adults in the UK classified as obese increased from 15% in 1993 to 26% in 2016^2^. The incidences of obesity-related diseases have also increased^3–5^. As obesity is a risk factor for developing gallstones^6^, surgeons are encountering increasing numbers of obese patients requiring LC.

LC in obese patients is perceived to be more challenging and associated with higher conversion and morbidity rates. This may cause practitioners to unnecessarily defer or delay care in obese patients due to increased perioperative risk.

When laparoscopic surgery is performed on obese patients, it can be associated with increased intra-operative risks from anaesthetic complexities and technical difficulties. Although early reports suggested that important operative and outcome parameters showed no statistical differences between the obese and non-obese^7,8^, others reported higher rates of postoperative complications such as wound infection, chest infection, atelectasis and deep vein thrombosis (DVT)^9^.

The increased body mass and metabolic demands also result in cardiovascular and pulmonary adjustments, increasing the risk of cardiac failure, arrhythmias and airway compromise resulting in intraoperative hypoxia. Such effects are exaggerated in the supine position and in conjunction with pneumoperitoneum^10,11^.

Access was perceived to be one of the main difficulties in LC in obese patients. Increased abdominal wall thickness and extra peritoneal fat^10,11^ can pose a challenge to safe peritoneal entry and failure to establish pneumoperitoneum may lead to higher conversion rates^12^. Suboptimal access techniques will make maintaining pneumoperitoneum a challenge. Gallbladder retraction may be hindered by a heavy liver and a hanging left lobe may obscure the cystic pedicle. Retraction of the omentum and the duodenum may require extra measures. Excess fat on the cystic pedicle increases the difficulty in dissecting and separating the cystic structures. Camera port closure requires careful attention and can occasionally be difficult unless access was established in an optimal way and the surgeon’s closure technique is skilful.

Postoperative deep vein thrombosis (DVT) or pulmonary emboli were reported in 5–12% of obese patients^13^ necessitating adequate pharmacological and mechanical preventive measures.

The primary aim of this study was to determine whether the operative difficulty grading of performing LC is influenced by obesity. The secondary aims were to study how to overcome difficulties that are specific to obese patients, particularly when performing urgent biliary procedures during the index admission, and to detect any adverse obesity-related outcomes compared to a control cohort of non-obese patients.

## Materials and Methods

Analysis of a database of consecutive LCs and LCBDEs performed between August 2002 and Mach 2020 was conducted. The World Health Organisation definition of obesity (BMI 30 or greater) would include large numbers of patients undergoing LC. We have, therefore, defined obesity as a BMI of 35 or over, for the purpose of this study. Two groups were identified based on the BMI. Individuals with a BMI ≤ 34 were not considered obese. Individuals with a BMI ≥ 35 had class II obesity and those with a BMI ≥ 40 or ≥ 35 with obesity-related medical conditions; diabetes, hypertension or ischaemic heart disease had class III obesity, described by some authors as grossly obese^12^. In this institution, all such patients, whether elective or emergent, are referred to the subspecialised biliary unit while small numbers of elective cholecystectomies are performed by one and occasionally two other general surgeons. Patient’s demographics, type of admission, radiological findings, operative difficulty grade of LC on the Nassar scale^14,15^, operative time, open conversion, peri-operative complications and mortality were extrapolated. All emergency biliary patients are managed during the index admission^16^. No patient admitted as an emergency, was fit for general anaesthesia, and was denied surgery based on their high BMI. Very few elective outpatients with extreme obesity were referred for bariatric surgery prior to or combined with LC at another centre.

## Pre-operative Anaesthetic Workup of Morbidly Obese Patients

Elective patients undergo pre-operative assessment according to hospital policy. Liver reducing diets were not used at this unit. Emergency biliary admissions are assessed by the anaesthetists and prepared for index admission surgery except for occasional cases requiring cardiorespiratory assessment. Occasional patients require postoperative high dependency care but most are nursed on normal surgical wards.

## Operative Technique

All procedures were performed by the senior author or his trainees under direct on table supervision. Patients are securely positioned on the operating table using appropriate straps with careful attention to pressure areas.

LCs and LCBDEs were carried out with a standard four port technique in the American position. The epigastric port is inserted in the midline, entering the abdominal cavity to the right of the falciform ligament. Extra ports are rarely needed.

Modified open access was carried out to insert the 12 mm infra-umbilical, making the skin incision, clearing the subcutaneous fat and picking up the umbilical tube and the linea alba using two tissue forceps and making a fascial incision between them. A blunt forceps is inserted through the peritoneum at 45° towards the right costal margin and used to stretch the fascial incision enough to insert the 10–12 mm port through the defect and into the peritoneal cavity. This allows a tight fit and avoids carbon dioxide leakage (Supplementary Video file 1). Any pre-existing umbilical defects are utilised to enter the peritoneal cavity without fascial incision. Other measures, e.g. formal Hasson technique, optical trocars or balloon cannulas, to secure the port are not used. Some patients required modified access due to the presence of scars of abdominal surgery. Pneumoperitoneum is established and three 5 mm ports are inserted under vision in a straight line from the skin to the peritoneum in the direction the instruments inserted through them will be used. An optimal direction of liver retraction is helped, very occasionally, by extra 5 mm ports to retract a heavy liver or a large redundant left lobe obscuring the duodenum and cystic pedicle. Distension of the duodenum or stomach is overcome with orogastric/nasogastric decompression or with the use of 30° scopes. When safe under-vision insertion of secondary ports is hindered by distended bowel, this can be achieved by pushing the tip of subcostal port trocar, as it points into the peritoneum, into the epigastric cannula, using it as a sheath. The right lateral port is then inserted into the subcostal cannula in the same way if necessary, the so-called port-in-port access.

The dissection instrument and technique is shown in Supplementary Video file 2. Neither diathermy hooks nor endo-clips were used in this series, metal clips having been abandoned 23 years ago in favour of intracorporeal 2/0 Vicryl ties to secure the cystic duct and cystic artery. Cholangiography techniques have previously been published^17,18^. Gallbladder separation was carried out using the duckbill dissector achieving haemostasis in the process (Supplementary Video file 2) before placing the gallbladder in a retrieval bag, swapping the 10 mm scope with a 5 mm scope inserted into the epigastric port to allow under-vision retrieval and closure (Supplementary Video file 3A). The fascial defect of the 12 mm infraumbilical port is picked up and lifted, closer to the incision, using two blunt hooks then closed using a 0 Polysorb suture on a 5/8 circle needle under laparoscopic vision (Supplementary Video file 3B)^19^. The techniques used for LCBDE and the indications and methods of biliary drainage have previously been described^20^.

All patients received prophylactic antibiotics and mechanical antithrombotic measures at induction of anaesthesia. Postoperative prophylactic LMWH is given in the evening of the operation and patients with specific risk factors continued on LMWH for 1 to 2 weeks subject to the indication.

## Follow-up

Most patients undergoing LC were followed up within 2 months. Although later in the series, some telephone follow-up was conducted, and routine follow-up was discontinued as the healthcare system allowed patients who had any issues to be referred back to the unit by their general practitioners. The “number of episodes” was defined as the total number of biliary admissions until complete resolution, including previous admissions, the index episode and readmissions. “Total hospital stay” was calculated for all admissions even for day-case LC patients who had previous biliary episodes. “Presentation to resolution” was defined as the interval between elective or emergency admission and discharge to the community with complete resolution of symptoms or complications following biliary surgery.

## Statistical Analysis

Qualitative data were given as frequency and percentages. For continuous data, median and interquartile range were used and *p* value was calculated using unpaired Student *t* test. For categorical variables, *p* values and odds ratio with 95% confidence interval were calculated using Pearson uncorrected chi-square test. *p* value of < 0.05 was considered to be statistically significant. GraphPad Prism version 9.0.2 was used to calculate statistics.

Informed consent was obtained from all patients. The database was registered in the clinical audit department but no IRB approval was necessary for database analysis as all patients were treated according to standard protocols consistent with national and international guidelines.

## Results

**Fig. 1:**
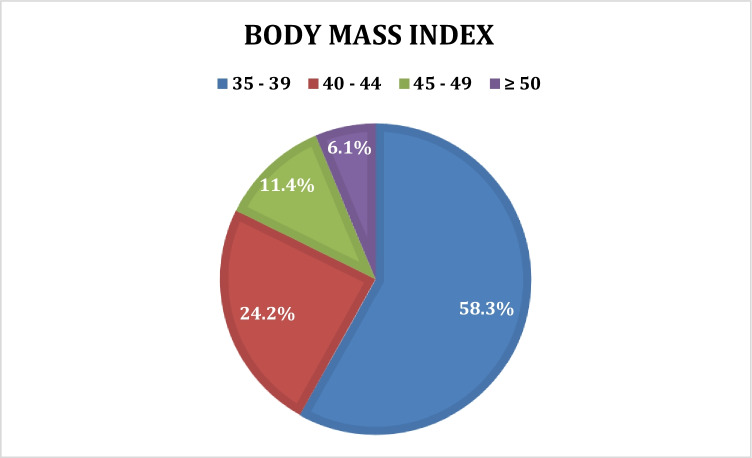
BMI distribution in the obese group. Median 38, mean 39.9

Of the 4699 patients in the study, 683 were identified as grossly obese with a BMI ≥ 35. A BMI ≥ 40 was recorded in 41.7%. Patients with a BMI of > 40 or a BMI of ≥ 35 with co-existing obesity-related complications (433, 63.4%) met the criteria of morbid obesity. The distribution of BMIs in the obese group is shown in Fig. 1. The obese group had significantly more females and was younger than the non-obese. An ASA classification of 3 or higher was recorded in 16.6% of the obese patients (Table 1).

**Table 1:** Demographic characteristics in obese patients and the non-obese group

	Obese *n* = 683 (14.5%)	Non-obese *n* = 4,016 (85.5%)	*p* value	OR (95% CI)
Sex				
Male	105 (15.4%)	1,143 (28.5%)	** < 0.001**	0.457 (0.367, 0.568)
Female	578 (84.6%)	2,873 (71.5%)		
Median age (years)	45 (IQR 35–55)	52 (IQR 39–65)	** < 0.001**	
ASA classification				
1	148 (21.7%)*	1,677 (41.8%)	** < 0.001**	0.386 (0.318, 0.468)
2	411 (60.2%)	1,679 (41.8%)	** < 0.001**	2.103 (1.782, 2.482)
3	109 (16%)	545 (13.6%)	0.069	1.209 (0.967, 1.513)
4	4 (0.6%)	14 (0.3%)	0.354	1.684 (0.553, 5.131)
Not recorded	11 (1.6%)	101 (2.5%)	0.152	0.635 (0.339, 1.189)

*ASA* American Society of Anaesthesiologists

^*^Although 21.7% of the obese population were recorded as having an ASA class 1, the current American Society of Anesthesiologists suggests that all patients with a BMI 30–40 are ASA class 2 (https://www.asahq.org/standards-and-guidelines/asa-physical-status-classification-system, accessed 2/12/2021). The original data was not altered in order to preserve data integrity

Although the two groups had equal proportions of elective and emergency admissions, the obese group was significantly less likely to present with certain gallstone complications, e.g. cholangitis and jaundice (Table 2). They also had a significantly lower incidence of CBD stone risk factors: deranged liver function tests or dilated bile ducts on ultrasound scans. Previous admissions with acute biliary episodes were recorded in equal percentages. However, 60.4% and 61.8% of the previous admissions occurred at other units or hospitals before the patients were referred to the biliary firm.

**Table 2:** Preoperative data in the obese versus non-obese group

	Obese *n* = 683	Non-obese *n* = 4,016	*p* value	OR (95% CI)
Urgency				
Elective	368 (53.9%)	2,018 (50.2%)	0.079	1.157 (0.983, 1.361)
Emergency	315 (46.1%)	1,996 (49.7%)	0.084	0.866 (0.736, 1.019)
Not recorded	0	2 (0.05%)	0.560	
Clinical presentation*				
Chronic biliary symptoms**	362 (53.0%)	2,932 (73.0%)	** < 0.001**	0.417 (0.353, 0.492)
Acute biliary pain	237 (34.7%)	1,508 (37.5%)	0.154	0.884 (0.746, 1.048)
Acute cholecystitis	62 (9.1%)	444 (11.1%)	0.123	0.803 (0.608, 1.062)
Acute pancreatitis	62 (9.1%)	379 (9.4%)	0.766	0.958 (0.723, 1.270)
Acute cholangitis	3 (0.4%)	126 (3.1%)	** < 0.001**	0.136 (0.043, 0.429)
Jaundice	111 (16.3%)	935 (23.3%)	** < 0.001**	0.639 (0.515, 0.794)
CBD stone risk factors	204 (29.9%)	1,852 (46.1%)	** < 0.001**	0.498 (0.418, 0.593)
Previous surgery	195 (28.6%)	1,565 (39.0%)	** < 0.001**	0.626 (0.524, 0.748)
Previous biliary admissions	81 (11.9%)	558 (13.9%)	0.151	0.834 (0.650, 1.069)
Preoperative MRCP	25 (3.7%)	281 (7%)	**0.001**	0.505 (0.333, 0.767)
Preoperative ERCP	10 (1.5%)	113 (2.8%)	**0.041**	0.513 (0.267, 0.985)

*CBD* common bile duct, *MRCP* magnetic resonance cholangiopancreatography, *ERCP* endoscopic retrograde cholangiopancreatography

^*^More than one diagnosis was recorded in some emergency admissions but only chronic biliary pain** in elective cases

The cholecystectomy operative difficulty grade, apart from grade 3, and the need for division of adhesions to the gallbladder were evenly matched in both groups. However, dissection of the cystic pedicle was significantly easier in the obese group (*p* = 0.002). Obesity was not associated with an increase in the very difficult (grade IV) or complex (grade V) LC. The incidence of LCBDE in the obese group was significantly lower (17.3% vs. 25.5%), with transcystic exploration (TCE) being equally successful in both groups. The median operative time, both for LC and LCBDE, was comparable in both groups, as were the rates of fundus first dissection, gallbladder perforation and the use of abdominal drains (Table 3). No open conversions occurred in the obese group. The complication rate was comparable in the two groups. There were no anaesthetic or access-related complications in the obese group. The only potentially obesity-related postoperative complication was wound infection; 13 (1.9%) occurred in the obese cohort compared to 29 (0.7%) in the non-obese (*p* = 0.002). Only one port site incisional hernia occurred in the morbidly obese as compared to 12 in the non-obese; however, the difference was not statistically significant (*p* = 0.483, OR 0.489, 95% CI 0.064, 3.769). Supplementary data file 1 details the complications requiring medical, radiological or surgical remedial reintervention measures or longer hospital stay occurring in both groups. The total complications rate and 30-day readmission rate are shown in Table 3.

**Table 3:** Operative parameters and technique in the obese versus the non-obese group

	Obese *n* = 683	Non-obese *n* = 4,016	*p* value	OR (95% CI)
Procedure				
LC	565 (82.7%)	2,991 (74.5%)	** < 0.001**	1.641 (1.329, 2.029)
LCBDE	118 (17.3%)	1,025 (25.5%)		
LC difficulty grade				
Grade 1	209 (30.6%)	1,319 (32.8%)	0.247	0.902 (0.756, 1.075)
Grade 2	200 (29.3%)	1,186 (29.5%)	0.895	0.988 (0.827, 1.181)
Grade 3	170 (24.9%)	788 (19.6%)	**0.002**	1.357 (1.122, 1.642)
Grade 4	94 (13.8%)	628 (15.6%)	0.209	0.861 (0.682, 1.088)
Grade 5	10 (1.5%)	95 (2.4%)	0.141	0.613 (0.318, 1.183)
Adhesiolysis*				
Gallbladder	434 (63.5%)	2,631 (65.5%)	0.318	0.918 (0.775, 1.086)
Hepatic flexure	119 (17.4%)	836 (20.8%)	**0.042**	0.803 (0.649, 0.992)
Duodenum	324 (47.4%)	2,052 (51.1%)	0.077	0.864 (0.734, 1.016)
Distant	66 (9.7%)	481 (12.0%)	0.081	0.786 (0.599, 1.031)
Calot’s triangle				
Normal	572 (83.7%)	3,284 (81.8%)	0.214	1.149 (0.923, 1.429)
Easy	328 (48.0%)	1,670 (41.6%)	**0.002**	1.298 (1.103, 1.527)
Accessory artery	230 (33.7%)	1,197(29.8%)	**0.042**	1.196 (1.006, 1.421)
Cystic duct stones	133 (19.5%)	689 (17.2%)	0.141	1.168 (0.950, 1.435)
Wide cystic duct	90 (13.2%)	571 (14.2%)	0.469	0.916 (0.721, 1.163)
IOC successful	682 (99.9%)	4,004 (99.7%)	0.483	2.044 (0.265, 15.745)
IOC abnormal	139 (20.4%)	1,130 (28.1%)	** < 0.001**	0.653 (0.535, 0.796)
Gallbladder condition**				
Chronic	459 (67.2%)	2,668 (66.4%)	0.694	1.035 (0.871, 1.230)
Hartmann’s pouch stones	111 (16.3%)	525 (13.1%)	**0.025**	1.290 (1.032, 1.613)
Contracted	73 (10.7%)	526 (13.1%)	0.081	0.794 (0.613, 1.029)
Empyema	55 (8.1%)	354 (8.8%)	0.514	0.906 (0.674, 1.219)
Acute	32 (4.7%)	218 (5.4%)	0.424	0.856 (0.586, 1.253)
Mucocele	42 (6.1%)	194 (4.8%)	0.145	1.291 (0.915, 1.821)
Dyskinesia/polyp(s)	3 (0.4%)	85 (2.1%)	**0.003**	0.204 (0.064, 0.647)
Mirizzi’s syndrome	9 (1.3%)	44 (1.1%)	0.611	1.205 (0.586, 2.481)
Fundus first dissection	16 (2.3%)	117 (2.9%)	0.406	0.799 (0.471, 1.357)
GB perforation/rupture	110 (16.1%)	665 (16.6%)	0.768	0.967 (0.776, 1.206)
Abdominal drain	325 (47.6%)	2,118 (52.7%)	**0.013**	0.814 (0.692, 0.957)
Open conversion	0	3 (0.06%)	0.475	
Median operative time (mins)				
LC	55 (IQR 40–70)	50 (IQR 38–65)	0.737	
LCBDE	100(IQR 70–132)	100(IQR 75–175)	0.751	
Complications	44 (6.4%)	328 (8.2%)	0.123	0.774 (0.559, 1.072)
Readmissions (included above)	21 (3.1%)	144 (3.6%)	0.502	0.853 (0.536, 1.358)
30-day mortality	1 (0.1%)	7 (0.2%)	0.870	0.840 (0.103, 6.836)

*LC* laparoscopic cholecystectomy, *LCBDE* laparoscopic common bile duct exploration, *IOC* intraoperative cholangiogram

^*^Division of adhesions between the gallbladder and more than one viscus was frequently recorded

^**^More than one gallbladder condition was entered in some cases

The median total hospital stay, including all admission episodes, and the number of biliary admission episodes were comparable in the two groups. The hospital stay of 3 and 4 days reflected the fact that nearly half of all patients were admitted as emergencies, the majority under the care of other firms, and had undergone initial treatment and diagnostic imaging prior to referral to the biliary firm. In addition, 17% and 25% underwent bile duct exploration, increasing their hospital stay. Most patients (85% and 82.9%) required only one admission for the resolution of their elective or emergency biliary episodes. The median presentation to resolution interval was 1 week in both groups (Table 4).

**Table 4:** Follow-up data, hospital stay, number of episodes and presentation to resolution intervals in morbidly obese vs. non-obese patients

	Obese (*n* = 683)	Non-obese (*n* = 4,016)	*p* value	OR (95% CI)
Number of episode(s)				
1	581 (85%)	3332 (82.9%)	0.174	1.169 (0.933, 1.466)
2	95 (13.9%)	588 (14.6%)	0.616	0.942 (0.746, 1.190)
3	4 (0.6%)	82 (2.0%)	**0.009**	0.283 (0.103, 0.774)
4	1 (0.1%)	11 (0.3%)	0.542	0.534 (0.069, 4.142)
5	2 (0.3%)	3 (0.1%)	0.106	3.929 (0.655, 23.554)
% documented follow-up	**(n = 513, 75.1%)**	**(n = 3,112, 77.5%)**		
Median total hospital stay (days)	3 (IQR 1–8)	4 (IQR 1–8)	0.065	
Median presentation to resolution (weeks)	1 (IQR 1–2)	1 (IQR 1–2)	0.053	

Data of the 683 obese patients were further analysed dividing them into two groups according to operative difficulty: 274 grades III, IV and V and 409 grades I and II. Some pre-operative criteria were predictive of difficult LC: male sex (21.5% vs. 11.5%), age (53 vs. 44 years), emergency admission (58% vs. 38.1%), jaundice (24.4% vs.10.7%) and an ultrasound scan showing a contracted or thick-walled gallbladder (28.4% vs. 1.9%) or dilated bile ducts (17.9% vs. 5.3%). (Supplementary data file 2). However, the difficult and the easy LC groups had matching BMI mean and median values. Operative and postoperative characteristics are shown in Supplementary data file 3. Increasing difficulty was significantly associated with indicators of delayed surgery in the obese: two or more admission episodes and total hospital stay.

## Discussion

The World Health Organisation definition of obesity includes patients with BMI 30 or greater^1^. This study contained such large numbers of patients with BMI between 30 and 35 that for the purpose of meaningful analysis, these were excluded and a BMI of 35 or higher was defined as “grossly obese.”

Grossly obese patients undergoing LC are perceived to present considerable anaesthetic and surgical challenges. This causes referring doctors and some surgeons to avoid elective LC resulting in patients suffering frequent painful episodes. Avoiding index admission LC for emergency admissions increases the risk of complications and repeat admissions.

In spite of the difficulties and risks, multiple studies have now shown the surgical benefits of LC in obese patients^7,11,21–24^. However, apart from the well-documented difficulties of access and pneumoperitoneum, no studies objectively examined whether obesity adds to the technical difficulty of LC. The current study reports the largest series of consecutive grossly obese patients undergoing LC using a validated descriptive operative difficulty grading system.

The significantly higher ASA 2 classification and a trend towards higher ASA 3 in the obese are expected on account of the associated co-morbidity and is consistent with the reported literature. A policy of index admission surgery for all comers with biliary emergencies who are fit for anaesthesia resulted in 85% of the obese group and 83% in the non-obese having definitive treatment of gallstone disease in a single episode. 11.9% vs. 13.9% had had previous biliary admissions for various reasons but only 40% of these under the biliary team. The substantial improvement in the quality of life resulting from this policy is reflected in a median presentation to resolution interval of 1 week in over 75% of patients.

## Pneumoperitoneum

Hussein et al. suggested that difficulty inserting a Veress needle at the umbilicus can be encountered in 25% of patients. The needle was introduced via a sub-costal stab incision instead^12^. The authors reported that attempted cutdown for direct introduction of the port also failed, leading to open conversion. Their assertion that inserting the cannula by open access can be virtually impossible is not supported by our findings. The standard modified open access technique used in this study was not associated with access-related complications.

The thickness of the abdominal wall was suggested by some authors to pose a challenge to the insertion and use of secondary ports^12,25^. Longer instruments may be useful but were not used in this series. Insertion at carefully selected positions and in the direction of the cannulas’ intended use can overcome the challenges of abdominal wall thickness, ensuring port stability during the manoeuvring and exchange of instruments. A standard access technique helps to overcome difficulties with occasional measures such as decompressing the stomach and duodenum, using 30° scopes, placing a port above the level of the umbilicus, utilising extra ports for the retraction of the duodenum or the transverse colon and its mesentery addressing specific difficulties in certain cases. The insertion of extra ports should observe the distance and direction to the target organ and 5 mm ports avoid the need for fascial closure.

## Liver Weight and Size

Metabolically healthy individuals who are obese have an increased risk of developing non-alcoholic fatty liver disease (NAFLD) compared with individuals of normal weight. The presence of metabolic abnormalities with obesity adds to that risk. The incidence of NAFLD increases with the number of metabolic abnormalities in overweight and obese patients^26^. However, this study was only concerned with the main perceived difficulty of LC in the obese, namely the size and weight of the liver which may interfere with retracting the gallbladder, and subsequently the liver, to display the cystic pedicle. Burnard et al.^27^ suggested that reducing liver size to improve access and facilitate dissection, reduced procedure complexity and operating time. In a randomised trial, a very low calorie diet (VLCD) significantly reduced pre-operative weight and operative time. Dissection of Calot’s triangle was significantly easier. Although this was a randomised controlled trial, it only included 21 vs. 25 patients and the assessment of difficulty used subjective criteria. Using operative time as a measure is subject to many variables other than liver size or indeed BMI. The current study showed no operative time differences between the obese and non-obese patients. While liver reducing diets have been advocated in patients undergoing elective LC, they are clearly not practical in the emergency setting and were not used during the current study.

Retraction of the gallbladder fundus is an essential step in displaying the cystic pedicle for dissection. Some authors addressed the role of optimal positioning of the lateral subcostal port^12^ at an adequate distance from the costal margin ensuring its insertion in the direction of retraction.

## The Cystic Pedicle

The amount of fat in the cystic pedicle may be greater in the grossly obese. However, it is interesting that dissection of the cystic pedicle was found to be significantly easier in the obese patients in this study. This may be the result of using blunt dissectors rather than the diathermy hook to incise the peritoneum and sweep most of the fat allowing the identification and isolation of the cystic structures.

While some pre-operative criteria were found to be significantly predictive of difficult LC, they were the classical predictors and none was specific to obese patients. The mean and median BMI in both groups were exactly the same.

Operative difficulty of LC in the obese is linked in most studies to body fat and is described using subjective technique-related criteria. Hussein et al.^12^ addressed the induction of pneumoperitoneum: the insertion of the lateral port, gallbladder fundus retraction and the closure of the fascial incisions at the end of LC. Simopoulos et al.^23^ and Paajanen et al.^28^ discussed the role of abdominal fat, fat in the cystic pedicle obscuring the anatomy, large fatty livers and a bulky transverse colon leading to dissection difficulties. Some authors attempted to classify difficulty according to gallbladder adhesions. Angrisani et al.^25^ used a simple scale for gallbladder adhesions: 0 for absent, 1 minimal, 2 mild and 3 massive. They also described the macroscopic appearances of the gallbladder with 12% of their patients having mucoceles, 12% empyemas and 4% Mirizzi syndrome. Ammori et al.^29^ used a similar system with gallbladder adhesions graded 0–3 and empyema reported in 8.8%. Burnard et al.^27^ used a reasonably detailed scale (1 very difficult, 2 difficult, 3 normal and 4 easy) used by the operating surgeon to grade the ease of Clot’s dissection, fundus retraction and liver displacement. However, all three elements seem to have been limited to the effects of the size of the liver on procedure difficulty.

**Fig. 2:**
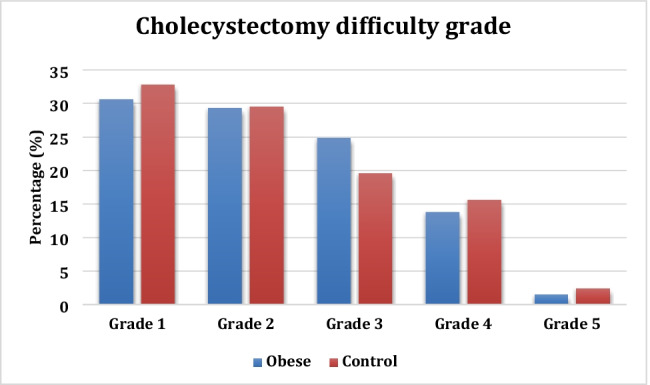
The distribution of cholecystectomy difficulty grades in the obese and contyrol groups of patients

The LC difficulty scale used in this series includes the access, cystic pedicle dissection and the condition of the gallbladder. This is a more objective method of difficulty grading which has been validated in large studies^30^. Only difficulty grade 3 was significantly more likely in the obese group. However, this trend was associated with acute cholecystitis, empyema of the gallbladder and Hartman’s Pouch stones, which are difficulty grade 3 by definition, in 54%. Grade 3 criteria that may be result from obesity were uncommon (6.5%) (Fig. 2).

Gallbladder extraction is carried out within retrieval bags by most authors with suction of bile, stone fragmentation and, if necessary, piecemeal removal of the gallbladder. Surgeons must resist extending the fascial incision to facilitate the extraction as wound extension adds to the difficulty of fascial closure, increases the operative time and is an important causative factor of port site incisional hernia^31^. Only one incisional hernia was recorded in obese patients in this series on long-term follow-up.

Difficulties of fascial closure have been addressed in many studies^12,28,32^. Hussein et al.^12^ described difficulties with closing the epigastric and infraumbilical port sites, highlighting the importance of closing fascial incisions to avoid incisional herniation and occasional bowel obstruction. They described picking up the edges of the fascial defect with Kocher’s forceps as the port and camera were removed. In the current series, a 5 mm telescope inserted through the 5 mm epigastric port allowed under-vision closure of the fascial defect. Nofal et al.^33^ reported BMI > 30 as a significant risk factor for port site incisional hernia on multivariate analysis. They suggested that patients should be warned of the risk during the consent process and that surgeons should close any fascial defect at port sites 10 mm or larger. This is consistent with the policy of avoiding 10–12 mm ports other than the periumbilical used in this series.

The median operative time in this study, both for LC and LCBDE, was comparable in both groups. This is in contrast to some recent studies^34,35^.

## Open Conversion

There were no conversions in our gross obesity cohort and only 3/4016 conversions (0.06%) in the non-obese. There are wide variations in the reported open conversion rates and the causes of conversion in different studies, subject to the experience of the surgeon and the inclusion of biliary emergencies, particularly acute cholecystitis. Hussein et al.^12^ reported one conversion (5%) resulting from failure to establish pneumoperitoneum in 20 grossly obese patients. Of 110 obese patients, Champault et al.^36^ reported a conversion rate of 4.5% compared to 1.8% in non-obese patients. However, some reports showed no significant differences^23^.

## Postoperative Complications

While similar rates of complications were reported^7,8,10,11^, some authors^28^ found greater postoperative morbidity in the morbidly obese (11.8%) and the obese (5.9%) than in the non-obese (4.7%). A comparison of various operative parameters and postoperative outcomes in a number of studies is shown in Table 5.

**Table 5:** Comparison of various studies addressing preoperative data and outcome parameters of LC in obese patients

Study	Year	BMI	Obese No (%)	Emergency %	IOC %	Operative time/min	Conversion %	Complications %
This study	2021	= or > 35	683 (14.6)	46.1	99.9	55	0	6.4%
Collet^37^	**1992**	** = / > 30**	**28 (9)**	**13.2**	**?**	**89**	**3.5%**	**3.5%**
Phillips^38^	1994	?	179 (21.3)	?	99.4	73	1.1	4.5
Gatsoulis^39^	**1999**	** = / > 30**	**23 (15.8)**	**20**	**-**	**95**	**0**	**4.3**
Ammori^29^	2001	= / > 30	205 (23.7)	6.3	73.6	80–102	4.4	9.7
Simopoulos^22^	**2005**	** = / > 35**	**94 (5.2)**	**N/A**	**-**	**45–50**	**7.4**	**2.1**
Chang^32^	2009	= / > 30	65 (10.3)	9.2	-	79 + / − 37.9	4.6	4.6
Paajanen^28^	**2012**	** = / > 30**	**437 (27.6)**	**15**	**22.4**	**84 + / − 40**	**11.7**	**12.5**
Tiong^23^*	2015	> 40	82 (9.5)	All elective	80	82	1.3	7.9
Tiong*	**2015**	**26–40**	**553 (69.2)**	**All elective**	**83**	**72**	**0.4**	**4.5**
Tandon^20^	2016	= / > 30	273 (47.8)	All elective	-	51	0	18.3

^*^The study by Tiong et al. classified BMI from 26 to 40 as obese in one group. This classification is unique in the literature. The paper also referred to the morbid/super obese group as having a BMI < 40, a mistake made repeatedly

Outcome parameters related to increased difficulty in the obese group were those directly resulting from delaying surgery, suggesting that such delay had no advantage. The reluctance to offer grossly obese patient LC in the index admission only results in a higher readmissions rate and an increased total hospital stay.

This study has some limitations. While consisting of relatively considerable cohorts, it spans 18 years. However, in spite of the discrepancy between the numbers of the obese and non-obese (control) groups, the inclusion of consecutive cases ensured the avoidance of selection bias. This is also a single surgeon series where subspecialist interest and expertise resulted from a practice with a high volume of emergency biliary cases and an unusually high proportion of patients with suspected bile duct stones. The experience, techniques and equipment have improved over time resulting in an inevitable adjustment of outcomes. It may be that the resulting expansion of indications and optimisation of outcomes cannot be generalised. However, most of the relevant technical points, e.g. access and closure, do not need advanced skill sets and are basic elements of LC. Highlighting their importance to overcoming difficulties should encourage surgeons without a subspecialist interest.

## Conclusions

LC can be safely performed on grossly and morbidly obese patients without increased operative difficulty or peri-operative complications. Standardising modified open access for the insertion of the camera port, at or immediately below the umbilical tube, is key to avoiding difficulties establishing pneumoperitoneum. Avoiding epigastric ports larger than 5 mm obviates the need for a usually difficult closure. Careful positioning of the working ports optimises the technical steps and reduces reliance on long or extra cannulas and instruments. This helps to overcome the reported and perceived difficulties during pedicle dissection, cholangiography or gallbladder separation. The adoption of blunt hook retraction to bring the fascial incision closer to the skin and the use of 5/8th circle needles facilitate adequate under-vision closure and reduce the risk of incisional herniation. Accessory ports must only be removed under vision after closure is achieved and checked and a second look of the pedicle and the gallbladder bed is completed.

This large study is the first to prospectively classify the difficulties encountered during LC in obese patients using an objective operative difficulty grading system. This allowed reliable adjustment of outcomes according to the case mix. No significant differences in operative and postoperative outcomes were found between the obese and non-obese. Grossly obese patients can safely be operated on by reasonably experienced surgeons during the index emergency admission, reducing potential interval complications that result in multiple admissions.

## Supplementary Information

Below is the link to the electronic supplementary material.

Supplementary file1 (DOCX 153 KB)

Supplementary file2 (DOCX 19 KB)

Supplementary file3 (DOCX 17 KB)

Supplementary file4 (MOV 35989 KB)

Supplementary file5 (MOV 97398 KB)

Supplementary file6 (MOV 4250 KB)

Supplementary file7 (MOV 97648 KB)
