# Supplementary material for: Operative Difficulty, Morbidity and Mortality Are Unrelated to Obesity in Elective or Emergency Laparoscopic Cholecystectomy and Bile Duct Exploration
Source: J Gastrointest Surg. 2022 May 31;26(9):1863–72. doi: 10.1007/s11605-022-05344-7 (PMC9489587; doi:10.1007/s11605-022-05344-7)
Supplement: Supplementary file 1 — Supplementary file1 (DOCX 153 KB) [file 11605_2022_5344_MOESM1_ESM.docx]

| Suuplemental data file 1 : Operative and postoperative complications requiring medical, radiological or surgical remedial reintervention measures or longer hospital stay. | | | | |
| --- | --- | --- | --- | --- |
| Peri-operative Complications | **Treatment** | | **Total**  **(n = 201)** | **Clavien Dindo Classification** |
|  | **Obese**  **n = 24 (3.5%))** | **Control**  **n = 177 (4.4%)** |  |  |
| CBD injury | - | 2 biliary bypass | 2 | G3b |
| Small bowel perforation during adhesiolysis/ port insertion | - | 2 primary closure | 2 | G3b |
| Bleeding port site | - | 2 suture under local anaesthetic  3 diathermy | 5 | G3a |
| Surgical emphysema | - | 3 | 3 | G1 |
| Pneumothorax | 1 chest drain | 1 chest drain | 2 | G3a |
| Post operative unstable angina | 2 | 1 | 3 | G1 |
| Blood transfusion | - | 1 | 1 | G2 |
| Shingles | - | 1 | 1 | G2 |
| Post operative pyrexial of unknown origin | 1 | 4 | 5 | G2 |
| Post operative pancreatitis | 3 | 10 | 13 | G1 |
| Pancreatic pseudocyst | - | 1 Percutaneous drainage | 1 | G3a |
| Urinary retention/ infection | - | 14 | 14 | G2 |
| Post operative myocardial infarction | - | 1 needing ITU support | 1 | G4a |
| Post operative perforated duodenal ulcer | - | 1 re-laparoscopy | 1 | G3b |
| Stroke/ TIA | - | 3 | 3 | G2 |
| Pulmonary embolism | - | 1 | 1 | G2 |
| Chest infection | 2 | 24  1 re-ventilation | 27 | G2  G3b |
| Post operative jaundice | 1 glucagon | 2  4 Glucagon  1 ERCP | 8 | G1  G2  G3a |
| Post operative ileus | - | 2 | 2 | G1 |
| Bile leak | - | 13 settled  6 ERCP/ stenting  1 percutaneous drainage  1 relaparoscopy | 21 | G1  G3a  G3b |
| Retained stone | - | 1 Glucagon  14 ERCP | 15 | G2  G3a |
| Acute kidney injury, T Tube loss | 1 | 3 | 4 | G4a |
| Abdominal pain after removal of T-tube | - | 1 | 1 | G1 |
| Non specific abdominal pain | - | 3 | 3 | G1 |
| Retained T-tube/ transcystic tube / drain | - | 1 ERCP  4 re-laparoscopy | 5 | G3a  G3b |
| Blood clot in CBD | - | 1 alteplase treatment | 1 | G2 |
| Post operative collection | - | 5  1 Percutaneous drainage | 6 | G2  G3a |
| Failed ERCP stenting , Mirizzi Type II & III | - | 1 Re-laparoscopy  1 relaparotomy | 2 | G3b |
| Leaking cholecystocolic fistula | - | 1 relaparoscopy | 1 | G3b |
| Perforated bowel injury/ tumour | 1 relaparatomy | 1 relaparoscopy | 2 | G5 |
| Mesenteric ischaemia | - | 2 relaparotomy | 2 | G5 |
| Wound infection* | 13 | 29 | 42 | G2 |
| Incisional hernia | 1 | 12 (only 2 repairs) | 2 | G3b |
|  |  |  |  |  |
| TIA = Transient ischaemic attack, CBD = Common bile duct, ERCP = Endoscopic retrograde cholangio-pancreatography.  * p=0.002, OR 2.668(1.380,5.158). Wound infections probably under reported as some are treated in the community. | | | | |
