# Supplementary material for: Operative Difficulty, Morbidity and Mortality Are Unrelated to Obesity in Elective or Emergency Laparoscopic Cholecystectomy and Bile Duct Exploration
Source: J Gastrointest Surg. 2022 May 31;26(9):1863–72. doi: 10.1007/s11605-022-05344-7 (PMC9489587; doi:10.1007/s11605-022-05344-7)
Supplement: Supplementary file 2 — Supplementary file2 (DOCX 19 KB) [file 11605_2022_5344_MOESM2_ESM.docx]

| Supplementary data file 2: Preoperative and Operative Criteria Predictive of Difficult Cholecystectomies in 683 Obese Patients | | | | |
| --- | --- | --- | --- | --- |
|  | **Easy**  **Grade I, II, III**  **n = 409 (59.9%)** | **Difficult**  **Grade IV, V**  **n = 274 (40.1%)** | **P value** | **OR (95% CI)** |
| Male Sex | 47 (11.5%) | 59 (21.5%) | **<0.001** | 0.473 (0.311, 0.719) |
| Median Age (IQR) | 44 years (34 – 54) | 53 years (41 – 59) | **<0.001** | - |
| Emergency admission | 156 (38.1%) | 159 (58%) | **<0.001** | 0.446 (0.326, 0.609) |
| Clinical Presentation |  |  |  |  |
| Acute Cholecystitis | 1 (0.2%) | 61 (22.3%) | **<0.001** | 0.009 (0.001, 0.062) |
| Acute Pancreatitis | 38 (9.2%) | 24 (8.8%) | 0.813 | 1.067 (0.624, 1.823) |
| Jaundice | 44 (10.7%) | 67 (24.4%) | **<0.001** | 0.372 (0.245, 0.565) |
| Ultrasound scan findings |  |  |  |  |
| Thick/contracted gallbladder | 8 (1.9%) | 78 (28.4%) | **<0.001** | 0.050 (0.024, 0.106) |
| Bile Duct Dilatation | 22 (5.3%) | 49 (17.9%) | **<0.001** | 0.261 (0.154, 0.443) |
| Suspected Bile Duct Stones | 101 (24.7%) | 103 (37.6%) | **<0.001** | 0.544 (0.391, 0.759) |
| Previous ERCP | 1 (0.2%) | 9 (3.3%) | **0.001** | 0.072 (0.009, 0.573) |
| Acute Cholecystectomy /Empyema at operation | 1 (0.2%) | 86 (31.3%) | **<0.001** | 0.005 (0.001, 0.039) |
