# Supplementary material for: Operative Difficulty, Morbidity and Mortality Are Unrelated to Obesity in Elective or Emergency Laparoscopic Cholecystectomy and Bile Duct Exploration
Source: J Gastrointest Surg. 2022 May 31;26(9):1863–72. doi: 10.1007/s11605-022-05344-7 (PMC9489587; doi:10.1007/s11605-022-05344-7)
Supplement: Supplementary file 3 — Supplementary file3 (DOCX 17 KB) [file 11605_2022_5344_MOESM3_ESM.docx]

| Supplementary data file 3: Operative and postoperative characteristics in Difficult v.s. Easy Cholecystectomies in 683 Obese Patients | | | | |
| --- | --- | --- | --- | --- |
|  | **Easy**  **Grade I, II, III**  **n = 409 (59.9%)** | **Difficult**  **Grade IV, V**  **n = 274 (40.1%)** | **P value** | **OR (95% CI)** |
| Bile Duct Explorations | 42 (10.2%) | 76 (27.7%) | **<0.001** | 0.298 (0.197, 0.451) |
| Median Operative Time | 50 min (40 – 70) | 105 min (80 – 130) | **<0.001** | - |
| Surgery in Index admission | 364 (89%) | 217 (79.2%) | **<0.001** | 2.125 (1.388, 3.251) |
| Two or more episodes | 45 (11%) | 57 (20.8%) | **<0.001** | 0.471 (0.308, 0.720) |
| Median Total Hospital Stay | 2 days (1 – 6) | 8 days (5 - 15) | **<0.001** | - |
